# Molecular Epidemiology of *Helicobacter pylori* Infection in a Minor Ethnic Group of Vietnam: A Multiethnic, Population-Based Study

**DOI:** 10.3390/ijms19030708

**Published:** 2018-03-01

**Authors:** Tran Thanh Binh, Vo Phuoc Tuan, Ho Dang Quy Dung, Pham Huu Tung, Tran Dinh Tri, Ngo Phuong Minh Thuan, Le Quang Tam, Bui Chi Nam, Do Anh Giang, Phan Quoc Hoan, Tomohisa Uchida, Tran Thi Huyen Trang, Vu Van Khien, Yoshio Yamaoka

**Affiliations:** 1 Department of Environmental and Preventive Medicine, Oita University Faculty of Medicine, Yufu-City, Oita 879-5593, Japan; bs.binh@yahoo.com.vn (T.T.B.); vophuoctuandr@gmail.com (V.P.T.); huyentrang110@yahoo.com (T.T.H.T.); 2 Department of Endoscopy, Cho Ray Hospital, Ho Chi Minh, Vietnam; quydung@gmail.com (H.D.Q.D.); quydung@gmail.com (P.H.T.); trantri73@gmail.com (T.D.T.); minhthuan1177@yahoo.com (N.P.M.T.); 3 Department of Endoscopy, Daklak Hospital, Daklak, Vietnam; quangpool@gmail.com; 4 Department of Endoscopy, Lao Cai Hospital, Lao Cai, Vietnam; namlaocaihp1@gmail.com; 5 Department of Endoscopy, Bach Mai Hospital, Hanoi, Vietnam; bsygiang@yahoo.com; 6 Department of Molecular Biology, 108 Military Central Hospital, Hanoi, Vietnam; phan_hoan2002@yahoo.com; 7 Department of Molecular Pathology, Oita University Faculty of Medicine, Yufu, Oita 879-5593, Japan; tomohisa@oita-u.ac.jp; 8 Department of Hepatogastroenterology, 108 Military Central Hospital, Hanoi, Vietnam; vuvankhien108@yahoo.com.vn; 9 Department of Medicine, Gastroenterology and Hepatology Section, Baylor College of Medicine, Houston, TX 77030, USA

**Keywords:** *Helicobacter pylori*, molecular epidemiology, virulence factor, Asian enigma

## Abstract

The *Helicobacter pylori*-induced burden of gastric cancer varies based on geographical regions and ethnic grouping. Vietnam is a multiethnic country with the highest incidence of gastric cancer in Southeast Asia, but previous studies focused only on the Kinh ethnic group. A population-based cross-sectional study was conducted using 494 volunteers (18–78 years old), from 13 ethnic groups in Daklak and Lao Cai provinces, Vietnam. *H. pylori* status was determined by multiple tests (rapid urease test, culture, histology, and serology). *cagA* and *vacA* genotypes were determined by PCR-based sequencing. The overall *H. pylori* infection rate was 38.1%. Multivariate analysis showed that variations in geographical region, age, and ethnicity were independent factors associated with the risk of *H. pylori* acquisition. Therefore, multicenter, multiethnic, population based study is essential to assess the *H. pylori* prevalence and its burden in the general population. Only the E De ethnicity carried strains with Western-type CagA (82%) and exhibited significantly lower gastric mucosal inflammation compared to other ethnic groups. However, the histological scores of Western-type CagA and East-Asian-type CagA within the E De group showed no significant differences. Thus, in addition to bacterial virulence factors, host factors are likely to be important determinants for gastric mucosal inflammation and contribute to the Asian enigma.

## 1. Introduction

Gastric cancer (GC) is the third most common cause of cancer-associated death and the fifth common cancer worldwide (IARC, GLOBOCAN 2012, http://globocan.iarc.fr/). Hence, the malignant disease remains a significant, serious public health problem, as well as a burden in both developed and developing countries. Interestingly, the incidence of GC fluctuates widely dependent on the differences in geography and ethnicity, such as between different continents, countries, and even regions belong to the same country [1,2].

The finding of Marshall and Warren about a spiral, Gram-negative, urease-producing bacteria colonizing in human gastric mucosa, called *Helicobacter pylori* (*H. pylori*) published in The Lancet in the 1980s, gave a crucial milestone toward understanding the pathogenic mechanism of upper digestive diseases [3]. The bacterial species infects half of the world’s population and a causal relationship between *H. pylori* and severe gastro-duodenal diseases was well-demonstrated through epidemiological and basic studies [4,5]. Intriguingly, despite *H. pylori*’s ubiquitous dissemination, the infection rate differs according to differences in countries, races, socio-economic statuses, and living or hygiene conditions [2,6]. On this basis, a hypothesis was given that a high incidence of GC might be attributable to a high prevalence of *H. pylori* infection and vice versa. However, it is not always applied in all cases, especially in the case of South Asia and Africa that were so-called, respectively, as Asian and African enigmas [7], from which these areas had a high infection rate, but low incidence of GC.

Practically, not all *H. pylori*-infected subjects develop such severe upper digestive tract diseases, but only 3% and 15% of them were estimated to suffer GC and peptic ulcer (PU) during their whole life, respectively [8]. This suggested that *H. pylori-*caused clinical progress is not only dependent on the bacterium, but is a combination of many factors including bacterial virulence, and host and environmental factors; or *H. pylori* has different virulent strains contributing to expressing different phenotypes [2].

The incidence of GC in Vietnam was classified as an intermediate risk in Asia, but the highest in Southeast Asia (age-standardized rate (ASR) of GC, 16.3/100,000 in both sexes). Several studies conducted in Vietnam initially disclosed an intimate association between *H. pylori* and gastroduodenal diseases [9,10]. Nevertheless, it should be noticed that Vietnam is a multiethnic nation consisting of 54 ethnicities and the previous studies only focused on the Kinh people, a major ethnic group accounting for 87% of total population of Vietnam [11]. The rest of the minor ethnic groups is usually scattered on high mountainous areas spreading from the north to the south and has not been studied yet. Due to the difference of *H. pylori* prevalence in various areas, as well as ethnic groups, an association between the microorganism and clinical outcomes from minor ethnic groups contribute to supply the essential information for gaining an overview of *H. pylori* prevalence, as well as the burden of *H. pylori*-caused diseases on the Vietnamese population. It is necessary to assist in establishing a feasible, practical screening strategy of GC in Vietnam. Moreover, it is particularly meaningful to minor ethnic groups which have many difficulties in contacting healthcare services due to their low socioeconomic and living conditions. Therefore, the aim of the study was to reveal the *H. pylori* infection in minor ethnic groups residing in mountainous areas, Daklak and Lao Cai, Vietnam, and investigate the virulence factor involved.

## 2. Results

### 2.1. Characteristics of the Study Population

**Figure 1:**
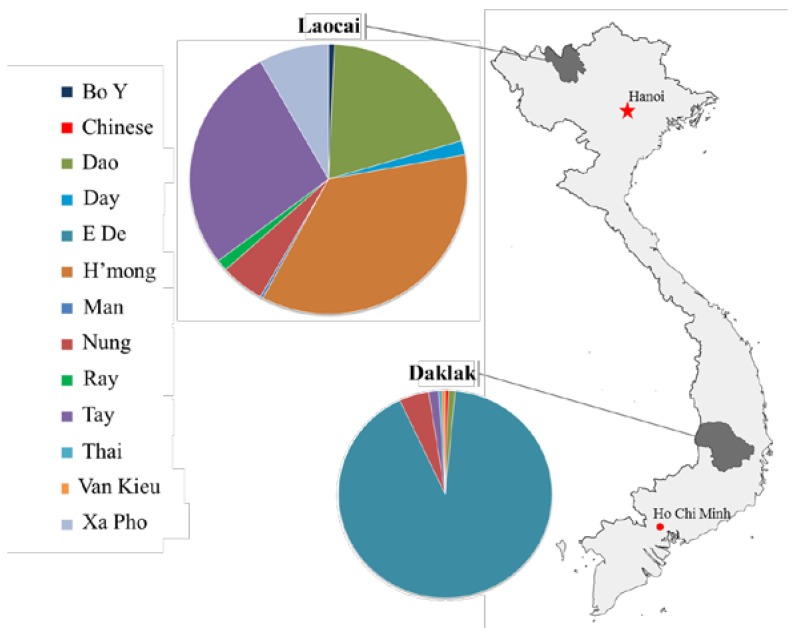
Map of Vietnam showing the geographical location and the frequency of the studied minority ethnic groups residing in Daklak and Lao Cai.

In total, 494 volunteers (210 males and 284 females), median age 38, interquartile range (IQR) 17 years old, were enrolled. Of which, there were 183 E De, nine Nung, three Tay, two Dao, one Van Kieu, one Chinese, one Thai in Daklak; and 105 H’mong, 15 Nung, 80 Tay, 58 Dao, 24 Xa Pho, five Day, four Ray, two Bo Y and one Man in Lao Cai province (Figure 1), of which the clinical manifestations included 465 (94.1%) with gastritis, 16 (3.2%) with gastric ulcer (GU), 12 (2.4%) with duodenal ulcer (DU), 19 (3.8%) with GERD, and 1 (0.2%) with GC.

### 2.2. The Prevalence of H. pylori Infection in General as Well as Its Differences between Geographical Regions and Ethnicities

In general, 188/494 (38.1%) were *H. pylori*-positive by culture, or at least two positive tests in the case of negative cultures. This infection rate in Daklak was significantly higher than that in Lao Cai (51.0% vs. 29.3%, *p* < 0.001). In a more detailed analysis, Table 1 shows that E De and Nung ethnicities in Daklak had significantly higher prevalence of *H. pylori* infection compared to Nung, Tay, and Dao ethnicities in Lao Cai.

Since the sample size is equal or smaller than 5, some ethnic groups, such as Tay, Dao, Van Kieu, Thai, Chinese, Day, Ray, Bo Y, and Man were excluded from the analysis.

### 2.3. Risk of H. pylori Infection

Table 2 presents the associations between *H. pylori* acquisition and potential risk factors, including age group, gender, geographical location, ethnicities, and marital, smoking, and drinking status. The results showed that subjects living in Daklak had a significantly higher risk of *H. pylori* infection than those in Lao Cai (crude OR, 2.52; 95% CI, 1.73–3.65). With respect to minor ethnic groups, E De (crude OR, 2.44; 95% CI, 1.64–3.62) was at significantly higher risk, whereas Tay (crude OR, 0.30; 95% CI, 0.16–0.56) and Dao (crude OR, 0.37; 95% CI, 0.17–0.73) were at significantly lower risk of *H. pylori* acquisition. 

To estimate the effect of each studied independent factor on the risk of *H. pylori* infection, as well as the complex interplay between them, multivariate logistic regression was performed. Among all the evaluated characteristics, the final model showed that people residing in Daklak (adjusted OR, 2.84; 95% CI,1.93–4.20), age group (adjusted OR, 1.79; 95% CI,1.05–3.07), and Xa Pho ethnicity (adjusted OR, 3.04; 95% CI,1.29–7.16) were significantly independent associated with the risk of *H. pylori* infection. 

### 2.4. H. pylori and the Gastroduodenal Diseases

Among *H. pylori*-infected participants, gastritis is the most prevalent diagnosis; 171/188 (91%), followed by GU 9/188 (4.8%), DU 8/188 (4.3%), and GERD 4/188 (2.1%). However, a high prevalence of *H. pylori* was observed in subjects suffering from GU (58.3%) and DU (64.3%), while subjects with gastritis were only 36.8% *H. pylori*-positive. When considering DU and GU as PU, the prevalence of *H. pylori* infection in PU was significantly higher than that in gastritis (60.7% vs. 36.8%, *p* = 0.02). The results from univariate analysis also showed that *H. pylori* positivity was significantly associated with PU (OR = 2.7, 95% CI 1.2–5.8). There were 19 cases with GERD diagnosed by endoscopic observation and no association with *H. pylori* infection was found.

### 2.5. H. pylori and Histological Evaluation

**Figure 2:**
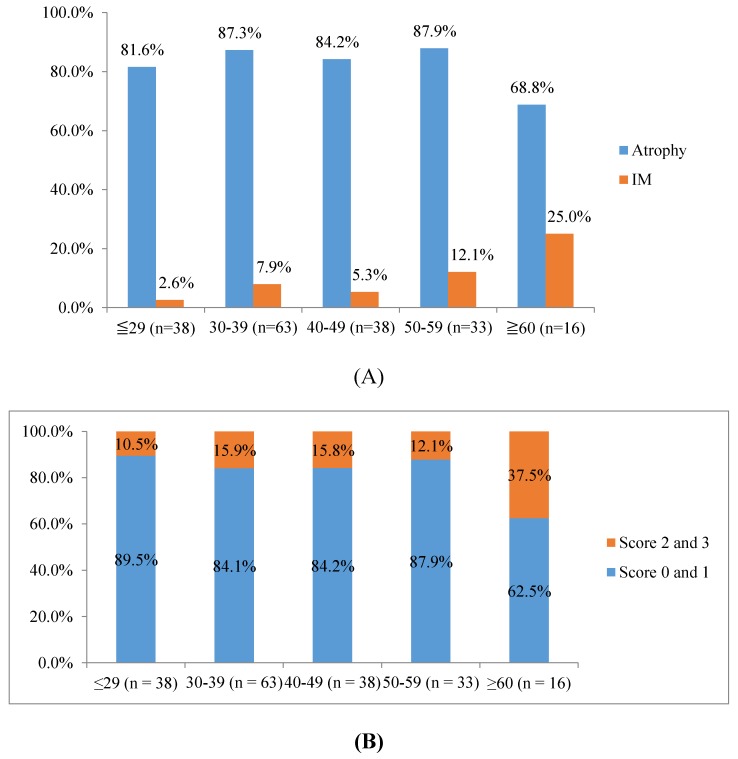
*H. pylori* and histology. (**A**) The distribution of atrophic gastritis (atrophy) and intestinal metaplasia (IM) based on age groups within *H. pylori* infected subjects. (**B**) The distribution of OLGA score (high risk: OLGA 2 and 3, and low risk: OLGA 0 and 1) based on age groups within *H. pylori*-infected subjects.

Among *H. pylori*-infected subjects, the intestinal metaplasia (IM) significantly increased with aging (*p* trend = 0.016) (Figure 2A), but no association was found in the case of atrophy. However, when the level of atrophic inflammation was classified by OLGA staging for gastritis criteria, our results showed that participants with high levels of atrophy, corresponding with an OLGA score of 2 or 3, significantly increased with aging (*p* trend = 0.017) (Figure 2B).

To comprehensively compare histological scores of *H. pylori* culture-positive subjects in different ethnicities, data from the Kinh people of a previous study were used (Table 3**)**. In our previous study, all populations studied were Kinh and there were 103 *H. pylori* culture-positive cases. Overall, the histological scores in E De were significantly lower than those in the remaining ethnicities (Table 3). Namely, the active (neutrophil) and chronic (monocyte) inflammation scores were significantly lower in E De compared to H’mong, Tay, Dao, and Kinh groups (all *p* < 0.05). Additionally, IM scores in the antrum in E De were significantly lower than that in H’mong, Tay, and Xa Pho groups; and the IM scores in the corpus in E De were also significantly lower than that in Kinh people (all *p* < 0.05). The OLGIM score in E De was significantly lower than that in H’mong, Tay, Xa Pho, and Kinh.

### 2.6. The Distribution of cagA and vacA Genotypes

The general frequency of *cagA* was 170/171 (99.4%). Among *cagA* positive strains, it was consisted of 97/170 (57.1%) East Asian-type CagA and 73/170 (42/9%) Western-type CagA (Table 4). Interestingly, Western-type CagA was only found in E De with high proportion, 73/89 (82%), whereas all the rest of minor ethnic groups, as well as the Kinh people, isolates possessed East Asian-type CagA, irrespective of geographical regions. Among 73 Western-type CagA strains (all from the E De group), 50 (68.5%), 15 (20.5%), 1 (1.4%), 1 (1.4%), and 6 (8.2%) were of ABC, ABCC, ABCCC, BC, and AB type, respectively. Out of 97 East Asian-type CagA strains, 90 (92.8%), 6 (6.2%), and 1 (0.01%) were of ABD, AB′BD, and AB type, respectively. Regarding the EPIYA motif, a total of 527 EPIYA motifs were obtained from the 170 *cagA* sequences. The distribution of EPIYA and EPIYA-like motifs were 490/527 (93%) EPIYA, 28/527 (5.3%) EPIYT, 6/527 (1.1%) ESIYA, and 2/527 (0.4%) and 1/527 (0.1%) ESIYT, respectively.

An association between CagA type and histological scores within ethnic groups was presented in Table 5. Within the E De group, there was no significant difference in histological findings between individuals infected with Western-type CagA and those infected with East Asian-type CagA. In addition, among subjects infected with East Asian-type CagA, the histological scores were also significantly lower in the E De group than in the other minor groups and the Kinh group.

All strains, 171/171 (100%), possessed *vacA* s1. In the m region, the distribution was 112/171 (65.5%) with m1, 57/171 (33.3%) with m2, and 2/171 (1.2%) with m1m2, of which *vacA* m1 was predominant in E De, 74/90 (82.2%), and Nung, 4/6 (66.7%), while *vacA* m2 was dominant in H’mong, 20/39 (51.3%), Tay, 7/12 (58.3%), Dao, 5/9 (55.6%), and Xa Pho, 7/11 (63.6%) (Table 4). When combining *cagA* and *vacA*, the frequency of East Asian-type CagA/*vacA* s1m1 in DU was significantly higher than that in gastritis (66.7% vs. 25.8%, *p* = 0.048) (Table 6).

## 3. Discussion

This is the first population-based cross-sectional study about *H. pylori* focusing on minor ethnic groups in Vietnam. Overall, the prevalence of *H. pylori* infection in our minor ethnic groups was 38.1%. The infection rate was lower than previous studies reported in Vietnam (56.1%–78.8%) [9,12,13]. The difference could be due to the difference in the study population (target population; minor ethnic groups, but not the Kinh ethnic group; general population, but not the hospital population), methodology (some studies used only serological test or rapid urine test). However, the result remained in line with the data reported by other countries in the Southeast Asia area, such as Thailand (23.3%–45.9%) [14,15], Laos (36.2%) [16], Myanmar (48%) [17], and Cambodia (approximately 30%, our unpublished data). Thus, it could be hypothesized that the prevalence of *H. pylori* infection in Vietnam might be not as high as previously reported.

Like many previous observational studies, the prevalence of *H. pylori* infection in the study differs from country to country, ethnicity to ethnicity, even in the same country [2,6]. Our study showed that the infection rate in Daklak (51%) was significantly higher than that in Lao Cai (29.3%) and also revealed a different risk of *H. pylori* infection between ethnicities (*p* < 0.001) (Table 2). Interestingly, the Nung ethnic group resided in both Daklak and Lao Cai; however the prevalence of *H. pylori* infection of Nung in Daklak was also significantly higher than that of Nung in Lao Cai (66.7% vs. 13.3%, *p* = 0.02). Indeed, the final model of multivariate analysis revealed that the risk of *H. pylori* acquisition is related to geographical regions, age groups, and ethnicities. Therefore, further studies based on the general population, and multicenter, multiethnic studies are necessary to generalize the *H. pylori* prevalence in the entire Vietnamese population, through which am accurate re-evaluation of the burden of *H. pylori*-caused disease can be made.

Our results indicated that *H. pylori* increased the risk of PU (OR = 2.7, 95% CI 1.2–5.8). In addition, *H. pylori*-infected people also increased the risk of precancerous lesions with aging, including intestinal metaplasia (*p* trend = 0.016) and severe atrophic status (*p* trend = 0.017). To remain in line with the Kinh ethnicity, as well as the global consensus, the microorganism infection is associated with severe gastroduodenal diseases and *H. pylori* eradication might contribute to decreasing the burden and risk of *H. pylori*-caused diseases [1,4].

It was fascinating that, in this study, the histological analysis of the gastric mucosal status exhibited that most histological scores in E De were significantly lower than those in other ethnicities, including the Kinh people (Table 3). According to the cascade pathway of GC proposed by Correa et al., gastritis is a crucial, indispensable step prior to developing GC and the progress is dependent on many factors, including bacterial virulence, and host genetic and environmental factors [4,18]. Many studies showed that a difference in *H. pylori* virulence factors might explain the variation in *H. pylori*-caused phenotype traits and we hypothesized that there was not much difference related to host and environmental factors for ethnicities residing in the same territory [2,9,19]. In efforts to clarify the difference, intriguingly only the E De ethnicity harbored Western-type CagA with high prevalence (82%), whereas all remaining studied ethnic groups harbored East Asian-type CagA. Indeed, many epidemiological studies showed that individuals possessing Western-type CagA exhibited lower gastric mucosal inflammation status and were more common in people suffering gastritis, but not in those suffering PU or GC compared to those possessing East Asian-type CagA [19,20,21]. Moreover, in vitro studies also showed that Western-type CagA expressed fewer biological activities, such as hummingbird phenotype, proinflammatory secretion, the disruption of cell junctions, or loss of cell polarity compared to East Asian-type CagA [22]. Thus, it is evident that Western-type CagA is less virulent than East Asian-type CagA. This might be the reason to explain why the gastric mucosal damage was milder in E De mainly harboring Western-type CagA compared to other ethnic groups harboring East Asian-type CagA. The presence of Western-type CagA in E De in Vietnam was similar to that in Okinawa, Japan [23]. To date, *H. pylori* has been used as a tool for tracing human migration, and further studies speculating where the E De came from is an interesting question.

Epidemiological and basic studies showed that *vacA* m1 is more virulent than *vacA* m2 [9,10,24]. In this study, the frequency of *vacA* m1 in the E De people was more predominant than other ethnic groups, however, the gastric mucosal damage was milder. This supported the hypothesis that it was better to study the combination or interaction between virulence factors rather than to find which factor is the most virulent [2]. Indeed, when combining *cagA* and *vacA*, the more virulent genotype East Asian-type CagA and *vacA* s1m1 presented a significant association with DU compared to gastritis. 

Although East Asian-type CagA is obviously more virulent than Western-type CagA [19,20,21], our results revealed that the histological scores between people infected with Western-type CagA and East Asian-type CagA within E De group were not significantly different (Table 5). Moreover, the histological scores in the E De group possessing East Asian-type CagA were also significantly lower than those in other ethnic groups with East Asian-type CagA. These implicated that although the bacterial factors are more or less virulent, the host susceptibility also plays an important role to modulating the level of gastric mucosal inflammation, irrespective of the virulent level of *H. pylori*. It suggested that the E De ethnicity has a somewhat specific susceptibility or host factor with a low risk of gastric mucosal inflammation that might be predisposed to a low risk of GC. Our finding was also similar to Thailand [25] and gave evidence to support a novel point of view in the Asian enigma that could be elucidated by specific host susceptibility apart from the presence of less virulent strains. Thus, further studies in the future in Southeast Asia area concentrating on the host factor are essential to verify the enigma.

## 4. Material and Methods

### 4.1. Study Design and Study Population

This is a population-based cross-sectional study to characterize the prevalence of *H. pylori* infection, as well as its virulence factors in a group of minor ethnicities in Vietnam. In the country, minor ethnic groups account for 13% of the general Vietnamese population and mainly reside in the highlands or mountains, especially in the northern mountainous regions and Tay Nguyen Central highlands. The survey was conducted in nine rural areas in Daklak, Tay Nguyen Central highland and Lao Cai Province, northern area, Vietnam from July 2012 to April 2013 (Figure 1). The distribution of minor ethnic groups was Kinh, 67%; E De, 17.2%; Nung, 4.1%; Tay, 3%; and other ethnic groups, less than 1% in Daklak. and Kinh, 35%; H’mong, 24%; Tay, 15.3%; Dao, 14.4%; Nung, 4.2%; Xa Pho 1.5%; and other ethnic groups less than 1% in Lao Cai, respectively (data available at http://www.gso.gov.vn/). Each area was sampled several times and at least two days per time was spent to recruit consecutive participants. The inclusion criteria were as follows: (1) age above 18 years old consenting to participate in the study; (2) no contraindication to upper gastroscopy; (3) no current treatment with proton pump inhibitors, antibiotics, bismuth-containing compounds, aspirin or nonsteroidal anti-inflammatory drugs within four weeks prior to the survey; and (4) no history of gastrectomy. Participants were volunteers in the community called to attend the study and would be given a health check-up, an endoscopy performed, and prescribed and given drugs. All steps were free of charge. All subjects were interviewed by trained medical staff to collect personal information about demographics, lifestyles, medical history, and so on.

Informed consent was obtained from all participants prior to the investigation. Ethical approvals were approved by the Ethics Committee of Daklak and Lao Cai Hospital, Vietnam and Oita University, Faculty of Medicine, Japan.

### 4.2. Sampling 

Four biopsy specimens (three from the antrum and one from the corpus) were taken during endoscopic procedure, of which two antral specimens were used for rapid urease test (RUT) and culture; and one antral and one corporal specimens were adopted for histological evaluation. PU, GC, and gastroesophageal reflux disease (GERD) were diagnosed under endoscopic observation and GC was confirmed by histopathology. Gastritis was defined in the absence of PU or suspicious malignancy in the stomach. After gastroscopy, blood samples were collected and then centrifuged to obtain serum. The obtained sera were stored at –80 °C until processing.

### 4.3. The Determination of H. pylori Status

To maximize the reliability in the detection ability of *H. pylori* infection, multiple tests comprising of RUT, culture, serological tests with enzyme-linked immunosorbent assay kit (Eiken Co., Ltd., Tokyo, Japan), and histology confirmed by immunohistochemistry (IHC) were used as previously described [9].

The gold standard of infection status was a positive culture. In the case of a negative culture, at least two positive tests among the following tests (histology, RUT, and serum-ELISA) were regarded as positive. If only one test was positive apart from culture, it was considered as undetermined *H. pylori* status. *H. pylori* was judged as negative when all test gave negative results.

### 4.4. Histological Status of Chronic Gastritis

All biopsy specimens were fixed in 10% formalin for 24 h and embedded into paraffin. A series of biopsy sections were stained with hematoxylin and eosin, and Giemsa. IHC using anti-*H. pylori* antibodies was performed as previously described [15]. Histological scores were evaluated based on the updated Sydney system (range 0–3: 0, none; 1, mild; 2, moderate; 3, marked) by an experienced pathologist (T.U) who would not know the sample identities [26]. In addition, the grading of chronic gastritis conveying information about anatomical extent of atrophic-metaplastic changes was also further assessed according to Operative Link for Gastritis Assessment (OLGA) and Operative Link on Gastric Intestinal Metaplasia (OLGIM) [27,28].

### 4.5. Cytotoxin Associated Gene A (cagA) and Vacuolating Cytoxotin (vacA) Genotype Analysis

An antral tissue specimen was used to isolate *H. pylori* strains by using standard culture method as previously described [29]. DNA extraction was performed by a commercial kit (QIAGEN DNeasy blood and tissue kit) according to manufacturer’s instruction. Extracted DNA was stored at –20 °C until used as a template for PCR.

*cagA* and *vacA* genotyping were performed as previously described [24,30]. Briefly, *cagA* was amplified using primers cagOMF (5′-AGC AAA AAG CGA CCT TGA AA-3′) and cagOMR (5′-AGT GGC TCA AGC TGC TGA AT-3′) and purified PCR products were sequenced using an AB 3130 genetic analyzer (Applied Biosystems, Foster City, CA, SUA). In silico, the DNA sequence was transformed into amino acids and aligned with MEGA v6 software [31]. Then, CagA types (East-Asian-type CagA and Western-type CagA) were defined according to flanking region of the EPIYA motif. Taken together, the strains possessing EPIYA-A, EPIYA-B, and EPIYA-C segments were considered as Western-type CagA and the strains possessing EPIYA-A, EPIYA-B, and EPIYA-D segments were considered as East Asian-type CagA. In case of untypable *cagA*, *cagA* status was confirmed with primers for the *cag* empty site. As for *vacA*, *vacA* s and m genotypes (s1 or s2, m1 or m2) were determined by the size of PCR products as previously described [24].

### 4.6. Statistical Analysis

Mean ± standard deviation or median was used to present the continuous variables. Frequency and percentage were used to present categorical variables. Data analysis was implemented with χ^2^ test, Fisher’s exact test, one-way ANOVA test, Mann–Whitney test, and multivariate logistic regression by using the SPSS software package (version 16.0; SPSS Inc., Chicago, USA). Regarding multivariate logistic regression analysis, independent factors with a significance of *p* < 0.25 were selected in the regression equation. A stepwise backward procedure was used to identify the best fit model. Odds ratio (OR) and 95% confidence interval (CI) were used to estimate the risk. The significance at *p* < 0.05 was established as statistically significant.

## 5. Conclusions

The prevalence of *H. pylori* infection varies based on geographical regions, age groups, and ethnicities, thus, nation-wide studies are necessary to evaluate the *H. pylori* prevalence in general populations, as well as the burden of *H. pylori* infection. In addition to *H. pylori* virulence factors, host factors might also play a crucial role responsible for gastric mucosal damage. This may be a novel insight to clarify the Asian enigma.

## Figures and Tables

**Table 1:** The prevalence of *Helicobacter pylori* infection in a population study, in general, and in Daklak and Lao Cai provinces, in particular.

	Ethnic	*H. pylori* Infection	Total
No. of participant (%)		188 (38.1%)	494
Daklak province			
	E De	94 (51.4%)	183
	Nung	6 (66.7%)	9
	Tay	0 (0%)	3
	Dao	0 (0%)	2
	Van Kieu	1 (100%)	1
	Thai	1 (100%)	1
	Chinese	0 (0%)	1
Lao Cai province			
	H′mong	40 (38.1%) ^a^	105
	Nung	2 (13.3%) ^a,b^	15
	Tay	15 (18.8%) ^a,b^	80
	Dao	12 (20.7%) ^a,b^	58
	Xa Pho	13 (54.2%)	24
	Day	1 (20%)	5
	Ray	2 (50%)	4
	Bo Y	0 (0%)	2
	Man	1 (100%)	1

^a^ indicates statistically significant differences when compared with E De at *p* < 0.05; ^b^ indicates statistically significant differences when compared with Nung in Daklak province at *p* < 0.05.

**Table 2:** Risk factors of *H. pylori* infection in the study population.

Risk Factor	*H. pylori* Positive/Total Number (%)	Crude OR	95% CI	*p*-Value
Age group				
≤ 29	38/118 (32.2%)	0.72	0.45–1.13	0.16
30–39	63/156 (40.4%)	1.15	0.77–1.73	0.49
40–49	38/107 (35.5%)	0.87	0.54–1.39	0.58
50–59	33/67 (49.3%)	1.70	0.98–2.95	0.06
≥ 60	16/46 (34.8%)	0.86	0.42–1.68	0.75
Gender				
Male	90/210 (42.9%)	1.42	0.98–2.05	0.06
Female	98/284 (34.5%)	1.00		
Geographical location				
Daklak	102/200 (51.0%)	2.52	1.73–3.65	<0.001
Lao Cai	86/294 (29.3%)	1.00		
Ethnicities				
E De	94/183 (51.4%)	2.44	1.64–3.62	<0.001
H′mong	40/105 (38.1%)	1.00	0.62–1.59	1.00
Nung	8/24 (33.3%)	0.81	0.29–2.04	0.67
Tay	15/83 (18.1%)	0.30	0.16–0.56	<0.001
Dao	12/60 (20%)	0.37	0.17–0.73	0.002
Xa Pho	13/24 (54.2%)	1.99	0.87–4.54	0.13
Other^*^	6/15 (40%)	0.64	0.24–1.68	0.5
Marital status				
Single	1/3 (33.3%)	1.00		
Married	187/491 (38.1%)	1.23	0.06–73.0	1.00
Smoking				
Yes	28/81 (34.6%)	0.84	0.49–1.41	0.53
No	160/413 (38.7%)	1.00		
Drinking				
Yes	55/121 (45.5%)	1.50	0.97–2.32	0.07
No	133/373 (35.7%)	1.00		

* include the ethnicities (including Day, Ray, Bo Y, Van Kieu, Thai, Chinese, and Man) with a sample size that is equal or smaller than 5.

**Table 3:** Comparison of histological scores between *H. pylori* infected ethnic groups.

		E De (n = 90)	Nung (n = 6)	H’mong (n = 39)	Tay (n = 12)	Dao (n = 9)	Xa Pho (n = 11)	Kinh (n = 103) ^a^
Antrum								
	Neutrophil	1.2 (1)	2.0 (2) ^b^	1.7 (2) ^c^	1.7 (2) ^d^	1.7 (2) ^e^	2.0 (2) ^f^	1.2 (1)
	Monocyte	1.6 (2)	2.3 (2.5) ^b^	2.1 (2) ^c^	2.2 (2) ^d^	2.0 (2)	2.2 (2) ^f^	1.7 (2)
	Atrophy	0.9 (1)	1.5 (1)	1.0 (1)	1.2 (1)	0.9 (1)	1.0 (1)	0.9 (1)
	IM	0.02 (0)	0.0 (0)	0.2 (0) ^c^	0.2 (0) ^d^	0.0 (0)	0.4 (0) ^f^	0.1 (0)
Corpus								
	Neutrophil	0.8 (1)	0.8 (1)	1.0 (1) ^c^	0.9 (1)	1.1 (1)	0.9 (1)	1.0 (1) ^g^
	Monocyte	0.6 (1)	0.5 (0.5)	1.2 (1) ^c^	1.1 (1) ^d^	1.2 (1) ^e^	0.6 (1)	1.5 (1) ^g^
	Atrophy	0.02 (0)	0.0 (0)	0.2 (0) ^c^	0.2 (0) ^d^	0.2 (0) ^e^	0.0 (0)	0.6 (1) ^g^
	IM	0.01 (0)	0.0 (0)	0.0 (0)	0.0 (0)	0.0 (0)	0.0 (0)	0.1 (0) ^g^
								
OLGA		0.9 (1)	1.5 (1)	1.1 (1)	1.2 (1)	0.9 (1)	1.0 (1)	1.0 (1)
OLGIM		0.03 (0)	0.0 (0)	0.2 (0) ^c^	0.2 (0) ^d^	0.0 (0)	0.4 (0) ^f^	0.2 (0) ^g^

^a^ Data obtained from our previous study. ^b^ indicates a statistically significant difference between E De and Nung. ^c^ indicates a statistically significant difference between E De and H’mong. ^d^ indicates a statistically significant difference between E De and Tay. ^e^ Indicates a statistically significant difference between E De and Dao. ^f^ indicates a statistically significant difference between E De and Xa Pho. ^g^ indicates a statistically significant difference between E De and Kinh. Statistically significant difference was determined on the basis of the Mann–Whitney test (*p* < 0.05).

**Table 4:** The distribution of *cagA* and *vacA* among minor ethnic group in Daklak and Lao Cai province.

*H. pylori* Culture Positive Cases	*cagA* Positive	CagA Type		*vacA* s and m	
		Western	East Asian	s1m1	s1m2
Daklak (n = 96)	95 (99%)	73 (76.8%)	22 (23.2%)	78 (81.3%)	18 (18.8%)
E De	89 (98.9%)	73 (82%)	16 (18%)	74 (82.2%)	16 (17.8%)
Nung^a^	4 (100%)	0 (0%)	4 (100%)	2 (50%)	2 (50%)
Van Kieu	1 (100%)	0 (0%)	1 (100%)	1 (100%)	0 (0%)
Thai	1 (100%)	0 (0%)	1 (100%)	1 (100%)	0 (0%)
Lao Cai (n = 75)	75 (100%)	0 (0%)	75 (100%)	34 (45.3%)	39 (52%)
H’mong	39 (100%)	0 (0%)	39 (100%)	17 (43.6%)	20 (51.3%)
Nung ^a^	2 (100%)	0 (0%)	2 (100%)	2 (100%)	0 (0%)
Tay ^a^	12 (100%)	0 (0%)	12 (100%)	7 (41.7%)	7 (58.3%)
Dao ^a^	9 (100%)	0 (0%)	9 (100%)	4 (44.4%)	5 (55.6%)
Xa Pho	11 (100%)	0 (0%)	11 (100%)	4 (36.4%)	7 (63.6%)
Ray	1 (100%)	0 (0%)	1 (100%)	1 (100%)	0 (0%)
Man	1 (100%)	0 (0%)	1 (100%)	1 (100%)	0 (0%)
Total (n = 171)	170 (99.4%)	73 (42.9%)	97 (57.1%)	112 (65.5%) ^b^	57 (33.3%) ^b^

^a^ These groups are present in both Daklak and Lao Cai provinces. ^b^ There were four cases with the genotype s1m1m2, and the cases were not listed.

**Table 5:** Comparison of histological scores between Western-type CagA and East–Asian-type CagA strains of different ethnic groups.

Cell Infiltration	Western-Type CagA E De (n = 73)	East Asian-Type CagA E De (n = 16)	East Asian-Type CagA Non-E De (n = 81)	East Asian-Type CagA Kinh (n = 103)
Histological scoresMean (median)				
Antrum				
Neutrophil	1.2 (1)	1.2 (1)	1.7 (2) ^a^	1.2 (1)
Monocyte	1.5 (2)	1.7 (2)	2.1 (2) ^a^	1.7 (2)
Atrophy	0.9 (1)	1.1 (1)	1.1 (1)	0.9 (1)
IM	0.03 (0)	0.0 (0)	0.2 (0) ^a^	0.1 (0)
Corpus				
Neutrophil	0.8 (1)	0.6 (1)	0.9 (1)	1.0 (1)
Monocyte	0.6 (1)	0.5 (1)	1.0 (1) ^a^	1.5 (2) ^a^
Atrophy	0.02 (0)	0.0 (0)	0.2 (0) ^a^	0.6 (1) ^a^
IM	0.01 (0)	0.0 (0)	0.0 (0)	0.1 (0)
OLGA	0.9 (1)	1.1 (1)	1.1 (1)	1.0 (1)
OLGIM	0.04 (0)	0.0 (0)	0.2 (0) ^a^	0.2 (0) ^a^

^a^ *p* < 0.05 by Mann–Whitney test when compared with Western type CagA E De.

**Table 6:** The association between *H. pylori* virulence factors and clinical outcomes.

	No. of Samples				
Type	GU	DU	PU	Gastritis	Total
No of culture positive cases	10	6	16	155	171
*cagA* positive	10 (100%)	6 (100%)	16 (100%)	154 (99.4%)	170 (99.4%)
Western-type CagA	4 (40%)	2 (33.3%)	6 (37.5%)	67 (43.2%)	73 (42.9%)
East-Asian-type CagA	6 (60%)	4 (66.7%)	10 (62.5%)	87 (56.1%)	97 (57.1%)
*vacA* s1	10 (100%)	6 (100%)	16 (100%)	155 (100%)	171 (100%)
*vacA* m1	8 (80%)	5 (83.3%)	13 (81.3%)	99 (63.9%)	112 (65.5%)
*vacA* m2	2 (20%)	1 (16.7%)	3 (18.7%)	54 (34.8%)	57 (33.3%)
*vacA* m1m2	2 (20%)	0 (0%)	2 (12.5%)	2 (1.3%)	2 (1.2%)
*vacA* s1m1	8 (80%)	5 (83.3%)	13 (81.3%)	99 (63.9%)	112 (65.5%)
*vacA* s1m2	2 (20%)	1 (16.7%)	0 (0%)	54 (34.8%)	57 (33.3%)
*vacA* s1m1m2	2 (20%)	0 (0%)	0 (0%)	2 (1.3%)	2 (1.2%)
Western-type CagA/*vacA* s1m1	4 (40%)	1 (16.7%)	5 (31.3%)	58 (37.4%)	63 (36.8%)
Western-type CagA/*vacA* s1m2	0 (0%)	1 (16.7%)	1 (6.3%)	9 (5.8%)	10 (5.8%)
East-Asian-type CagA/*vacA* s1m1	4 (40%)	4 (66.7%) ^a^	8 (50%)	40 (25.8%) ^a^	48 (28.1%)
East-Asian-type CagA/*vacA* s1m2	2 (20%)	0 (0%)	0 (0%)	45 (29%)	47 (27.5%)
East-Asian-type CagA/*vacA* s1m1m2	0 (0%)	0 (0%)	0 (0%)	2 (1.3%)	2 (1.2%)

^a^ *p* = 0.048 by Fisher exact test when compared between DU and gastritis.

## Abbreviations

GC	Gastric cancer
*Helicobacter pylori*	*H. Pylori*
PU	Peptic ulcer
ASR	Age-standardized incidence rate
DU	Duodenal ulcer
GU	Gastric ulcer
RUT	Rapid urease test
IHC	Immunohistochemistry
OLGA	Operative link for gastritis assessment
OLGIM	Operative link on gastric intestinal metaplasia
*cagA*	Cytotoxin-associated gene A
*vacA*	Vacuolating cytotoxin
OR	Odds ratio
CI	Confidence interval
IM	Intestinal metaplasia
